# Reducing bias and improving transparency in medical research: a critical overview of the problems, progress and suggested next steps

**DOI:** 10.1177/0141076820956799

**Published:** 2020-11-10

**Authors:** Stephen H Bradley, Nicholas J DeVito, Kelly E Lloyd, Georgia C Richards, Tanja Rombey, Cole Wayant, Peter J Gill

**Affiliations:** 1 Leeds Institute of Health Sciences, University of Leeds, Leeds LS2 9JT, UK; 2 Nuffield Department of Primary Care Health Sciences, University of Oxford, Oxford OX2 6GG, UK; 3 Institute for Research in Operative Medicine, Witten/Herdecke University, Alfred-Herrhausen-Straûe 50, 58448 Witten, Germany; 4 Centre for Health Sciences, Oklahoma State University, Tulsa 74107, USA; 5 Department of Paediatrics, University of Toronto, Toronto M5G 1X8, Canada

**Keywords:** Research and publication ethics, statistics and research methods

## Abstract

In recent years there has been increasing awareness of problems that have undermined trust in medical research. This review outlines some of the most important issues including research culture, reporting biases, and statistical and methodological issues. It examines measures that have been instituted to address these problems and explores the success and limitations of these measures. The paper concludes by proposing three achievable actions which could be implemented to deliver significantly improved transparency and mitigation of bias. These measures are as follows: (1) mandatory registration of interests by those involved in research; (2) that journals support the ‘registered reports’ publication format; and (3) that comprehensive study documentation for all publicly funded research be made available on a World Health Organization research repository. We suggest that achieving such measures requires a broad-based campaign which mobilises public opinion. We invite readers to feedback on the proposed actions and to join us in calling for their implementation.

## Introduction

Significant problems in medical research undermine efficient scientific discovery and efforts to achieve improved outcomes for patients. Across scientific disciplines, a diverse range of issues has resulted in a situation where published research findings frequently cannot be replicated (‘the reproducibility crisis’). Meanwhile, avoidable methodological failings and biases lead to ‘research waste’, which is estimated to account for 85% of all medical research funding.^1^

Many of these problems are rooted in incentives for individual medical researchers which are poorly aligned with the wider interests of science. Despite longstanding calls for ‘less research, better research, and research done for the right reasons’, concerns remain that quantity is valued over quality.^2^ Cultural problems in medical research are important in themselves but also impede progress in tackling biases in how research is reported and objectively presented.

This review introduces some of the most pervasive problems in medical research along with an overview of the current efforts to address these issues. We begin by describing some of the most significant problems related to research culture, reporting biases, and statistical and methodological issues. We then outline some important measures that have been instituted to address these problems. We conclude with a proposed strategy to help restore confidence in the reproducibility of medical research.

## Methods

This paper was informed by literature identified using a search strategy with the following terms: open science, open research, research quality, medic*, health*. Searches were conducted on CINAHL, EMBASE, MEDLINE and PsycINFO (Supplement 1).

## What are the problems in medical research?

### Research culture

#### Academic rewards system

Academics’ professional standing depends on demonstrating productivity through publication,^3^ with disproportionate rewards offered to those who attain publication in ‘luxury journals’ with high impact factors.^4^ Journal impact factors do little to capture the quality or value of individual research articles and can be manipulated.^5^ The extraordinary proliferation of research appears to reflect the pressure for academics to publish research rather than the development of genuine discovery which could lead to improved outcomes for patients.^6^

#### Conflicts of interests

Financial conflicts of interest have been shown to affect physician-prescribing habits, study conclusions and guideline recommendations.^7–10^ Potential competing interests have traditionally been regarded as monetary, but may also include professional, political or personal considerations.^11^ High-profile scandals have demonstrated undisclosed conflicts of interest undermine trust in the objectivity of research.^12–14^ While non-disclosure of competing interests does not necessarily affect assessments of study quality, authors’ interests can have a bearing on findings.^15^

Interest disclosures for published research are frequently incomplete.^16,17^ Conflict of interest recording and policies in institutions that host research are also poor, and in several cases journal editors, as well as researchers, have potential conflicts of interest.^18,19^ Voluntary declarations from the pharmaceutical industry have been criticised as inadequate, due to the ability of individuals to opt out.^20^ Meanwhile, other voluntary registers have limited coverage since they require eligible individuals to ‘opt in’. For example, a voluntary register of interests for doctors in the UK contains details of only 0.002% of all those registered with the General Medical Council.^21,22^ In several countries, ‘sunshine acts’ require disclosure of physicians financial interests, and calls have grown for similar requirements to be introduced to the United Kingdom.^23,24^

### Reporting biases

‘Reporting bias’ encompasses several sub-biases caused by selective disclosure or withholding of information, either intentionally or unintentionally, related to study design, methods and/or findings.^25^ While several types of reporting biases have been described, we will focus on two of the most widely studied: publication bias and spin.

Publication bias refers to the propensity of certain types of research to become published, while other types remain unpublished. This results in a distortion of the published record which disproportionately features findings that are deemed to be novel, striking or that provide evidence in favour of a proposed intervention.^26^ While publication bias is commonly understood to be driven by the perception that journals are unlikely to accept so-called ‘negative’ or ‘uninteresting’ results, researchers also perpetuate this bias by failing to submit such research. Publication bias has also been demonstrated to affect regulatory decisions and ultimately clinical practice.^26^ The problem appears to be culturally entrenched and, in some cases, conflicts of interest are implicated.^7,27^ Some journals have instituted initiatives to encourage publication of ‘negative’ findings to help remedy publication bias.^28,29^

Spin refers to the practice of distorting or misrepresenting results to appear more ‘positive’, newsworthy or interesting.^30^ While ‘spin’ falls short of outright falsification or fraud, experiments show that readers of studies with spin draw more favourable interpretations of interventions than when results are presented more objectively.^31^ Of particular concern is that spin in journal abstracts influences press releases, and studies that obtain press coverage receive greater numbers of citations.^32,33^ Spin manifests in a variety of ways including failure to mention study limitations, selective ordering and highlighting of outcomes, and drawing exaggerated inferences from study results.^34^ Spin appears to be increasingly common while peer review has proved insufficient to counter it.^35,36^ Reviewers who identify spin usually do not succeed in having it removed from manuscripts and some reviewers actually suggest the addition of spin.^37^

### Statistical and methodological issues

There is substantial debate throughout science about the role of *p* values in determining statistical significance.^38^ Reporting of *p* values has become much more common in recent decades, with 96% of papers containing *p* values of 0.05 or less.^39^ The ubiquity of published *p* values of <0.05 can be explained by the strong incentives for researchers to publish significant results.^40^ *p* values are also often misinterpreted and it has been estimated that most claims based on *p* values are false.^38,41^ Some scientists suggest that the threshold of significance for *p* values should be reduced or abandoned altogether, that greater emphasis be placed on effect sizes and confidence intervals, or that Bayesian statistics should be used to provide greater discriminatory utility when appropriate.^42,43^

The availability of modern statistical software means that, in the absence of an accessible protocol, it is relatively easy to generate statistically significant results through repeated analyses, a practice that has been dubbed p-hacking.^44^ P-hacking in turn facilitates so called HARKing (Hypothesising After the Result is Known), whereby researchers can retrospectively generate a matching hypothesis to a significant *p* value.^45^ Researchers may also selectively report statistically significant results or exploit ‘undisclosed flexibility’ in how analyses are conducted, allowing researchers to provide evidence for virtually any hypothesis.^46^

## Existing measures addressing problems in medical research

Several measures have been introduced to improve the reproducibility and transparency of medical research. Although no individual measure would be a cure-all, many successes have been documented. These highlighted achievements demonstrate that coordinated action to improve the research landscape is possible and necessary.

A number of important measures are considered in this article with some additional initiatives outlined in Table 1 and Table S1 in Supplement 2. It is beyond the scope of this review to survey all relevant actions that have been undertaken to address problems in medical research.

**Table 1.** Initiatives and organisations working to reduce waste and improve the openness and quality of research.

Category	Initiative/Organisation	Description	URL
Research culture	Sunshine UK	Voluntary register of doctors’ declared interests.	http://www.whopaysthisdoctor.org/
	Open Science Badges	Badges appended to publications to acknowledge and incentivise open science practices.	https://cos.io/our-services/open-science-badges/
	REWARD Alliance	Originated in 2014 *Lancet* series on waste in research. Promotes efforts to increase the value of research and reduce waste in research.	http://rewardalliance.net/
	San Francisco Declaration on Research Assessment (DORA)	Initiative which calls for improvement in how research quality is evaluated.	https://sfdora.org/
	UK Reproducibility Network (UKRN)	Initiative which promotes the practices of open science.	https://bristol.ac.uk/psychology/research/ukrn/
Reporting biases	AllTrials	Campaign to ensure all clinical trials are registered and published. Highlights problem of publication bias, e.g. through ‘unreported clinical trial of the week’ and trial trackers which monitor reporting performance.	https://www.alltrials.net/
	Enhancing the QUAlity and Transparency Of health Research (EQUATOR)	International network which promote transparent and accurate reporting and wider use of robust reporting guidelines.	https://www.equator-network.org
	TranspariMED	Campaigning organisation which advocates for registration and full reporting of clinical trials.	https://www.transparimed.org/
Improving methodological and statistical practices	Open Science Framework (OSF)	Online platform that facilitates open sharing and preregistration of research.	https://osf.io/
	Oxford – Berlin summer school on open research	Training for researchers organised by the QUEST Center for Transforming Biomedical Research and Reproducible Research Oxford.	https://www.medsci.ox.ac.uk/news/oxford-berlin-summer-school-2019
	Evidence-Based RESearch (EVBRES)	European network established to promote evidence based clinical research, particularly the need to use systematic reviews when planning new studies and when placing new results in context.	https://evbres.eu

### Research culture

Training in medical research, as in clinical medicine, is based largely around an apprenticeship model. Much attention has therefore been given to providing positive mentorship that promotes integrity.^47^ Several initiatives such as the UK Reproducibility Network have been established to promote values and practices in research conducive to the principles of open science within academic institutions.^48^

Several other measures have been proposed in order to align academic promotion criteria with responsible research practices. Suggestions include appraising research quality and reproducibility, and placing limits on the volume of publications that can be periodically submitted for institutional appraisal.^49–52^ The San Francisco Declaration on Research Assessment (DORA) and the Hong Kong Principles have been formulated improve the assessment of researchers while the Transparency and Openness Promotion guidelines (TOP) includes several standards by which to assess research and journals.^53–55^

### Reporting biases

#### Clinical trial registration

Many of the problems outlined above, such as publication bias, spin, HARKing and p-hacking occur after research has been conducted. ‘Pre-registering’ studies prior to collecting data establish a record of research scheduled to take place including the hypotheses, methods, analyses and outcomes. Researchers can then be held accountable to the methods and outcomes they prespecified and expected to justify any deviations.

Registration of clinical trials is promoted by the World Health Organization through a number of approved primary registries and has increasingly become an expectation of funders and publishers.^56,57^ The requirement by the International Committee of Medical Journal Editors is that all trials should be registered before being considered for publication and the passage of the FDA Amendments Act 2007 mandating registration in the USA were major milestones in achieving widespread trial registration.^58,59^ Mandatory registration requirements have in turn facilitated mandatory reporting requirements, with the expectation that full findings of all trials are disseminated in some form, be it through journal publication or alternate routes provided by regulatory bodies or trial registries.^60^ As well as from clinical trials, systematic reviews are also routinely registered prior to study commencement.^61^

Registration should be understood as one tool to help ensure researchers share their results and to discourage unjustified deviations from research plans. However, registration alone is not a panacea. For example, undeclared ‘outcome switching’ between registration and publication is common among registered trials.^62^ Journals could routinely ensure that reported outcomes match pre-specified registered outcomes. Despite these caveats, the value of registration should not be understated, since without it such deviations would not be detectable at all.^63,64^

#### Registration of non-trial research

Although registration is now an expectation for clinical trials and systematic reviews, it remains voluntary and is employed relatively infrequently for other research types. For example, observational studies would benefit from registration and pre-specification of hypotheses and methods. Free public platforms, such as the Open Science Framework, are a highly accessible means for all researchers to publish time-stamped protocols and analysis plans. We believe that registration or publication of a priori protocols should become an expectation, and justified when not present. Broad adoption of study registration and protocol publication could dissuade authors from presenting results from exploratory work as hypothesis-based research, combat p-hacking and HARKing, and create a permanent record of planned research that can mitigate publication and other reporting biases. ‘Registered Reports’ are a related initiative which is discussed below.

#### Publication checklists

One initiative employed to improve transparency and quality of reporting has been the widespread requirement by journals for authors to submit checklists along with manuscripts, indicating whether reporting standards have been met. Checklists now exist for all major study designs of which the Consolidating Standards of Reporting Trials (CONSORT) is perhaps the best known and endorsed by hundreds of journals.^65^ CONSORT has been associated with some improvement in completeness of reporting, but instances of poor CONSORT compliance remain.^63,66^ Even when discrepancies are clearly identified by journal readers, authors and journal editors infrequently make corrections.^64^ While the existing self-regulation of checklist compliance has yielded imperfect results, journal editors, or perhaps specially trained editorial assistants, could vet publications to ensure accurate reporting before publication.^67^

### Statistical and methodological issues

#### Education

The importance of high-quality training in statistics and open science methods are increasingly recognised.^68,69^ Several educational opportunities have been established, some of which are identified in Table 1 and Supplement 2. Although such training is welcome, only a small proportion of medical researchers will benefit from such initiatives with access limited in low- and middle-income countries.^70^ Therefore, it is vital that capacity to deliver such training is developed and maintained within academic institutions.

#### Data sharing

For research to be fully appraised and potentially reproduced, additional information beyond a study publication is required. Registrations can provide some additional detail, but in many cases, other documentation such as study protocols, analysis plans and individual patient data are necessary to understand and assess or reproduce the study. Making such data available would also allow other researchers to use the same data to answer different research questions.^71^ Unfortunately, research data are often not made available, even in summary forms or through secure mechanisms that would minimise the risks to breaches of patient privacy and confidentiality.^72–75^

The International Committee of Medical Journal Editors has instituted requirements for data sharing for published clinical trials. While such policies are welcome, research suggests that even with these requirements data are available in only about half of publications.^72^ Persuasive appeals have been made to institute greater data sharing for all study types, including the proposition that research papers become one of a series of ‘threaded documents’ with underlying data made available as a matter of course.^71,76^ Several data-sharing platforms are available to researchers.^77,78^

### How to improve medical research?

Significant gains in transparency have been achieved in clinical trial registration through the combined efforts of researchers, journals, funders, campaigners and legislators. Meanwhile continued attention has been brought to the ‘reproducibility crisis’ through the Open Science movement, which has connected international collaborators who share a determination to improve scientific practice through meta-scientific study. While it remains vital that emphasis is placed on improving statistical and ethical education for researchers and on addressing cultural issues within research, there is a need for immediate and definitive action to improve the quality of medical research.

We propose a strategy that includes three measures to achieve further improvements in the transparency of medical research. These measures are:

1. mandatory registration of interests for all people and institutions who conduct and publish health research;
2. all journals and funders support uptake of registered reports; and
3. publicly funded research is pre-registered and published on a World Health Organization-affiliated research registry.

Box 1 outlines how this strategy was developed, and Table 2 summarises some of the ways this could improve research.

**Table 2.** Problems in medical research and how they can be mitigated by authors’ proposed strategy.

Problem	Problem description	Relevant proposed solution(s)	How proposed solution(s) addresses problem
Publication bias	Tendency for results deemed ‘negative’ or ‘uninteresting’ to remain unpublished.	Registered Reports Research Registry	Study accepted for publication based on methods, not results Study results and documents made available, regardless of publication status
‘Spin’	Practice of presenting results of study as more striking, ‘positive’ or newsworthy than warranted.	Registered Reports Research Registry Mandatory Declaration of Interests	Reduced incentive to ‘spin’ to obtain publication Study documentation available to allow greater scrutiny of researchers’ claims Information on possible conflicts of interest allows peers to judge if researchers have vested interest in applying spin to study
Scientific fraud	Deliberate falsification of evidence, for example fabrication of results.	Research Registry	Availability of full study documentation allows peers to scrutinise results. Researcher compelled to demonstrate ‘not just the answer but their working out’
Non-adherence to reporting checklists	Inaccurate self-disclosure by researchers of fulfilment of checklist statements.	Research Registry	Peers can scrutinise methods from available study documentation
HARKing	Researchers generate hypotheses to fit results and present these as if formulated prior to obtaining results.	Registered Reports	Hypotheses and aims are agreed prior to undertaking research. Any further post hoc analyses are declared as such
P-Hacking	Researchers manipulate results until findings generated which satisfy statistical significance.	Registered Reports Research Registry	Analyses agreed prior to generation of results Analysis plans and code available to peers for scrutiny
Outcome switching	Researchers do not report certain outcomes, or switch primary and secondary outcome, to highlight favoured results.	Registered Reports Research Registry Mandatory Declaration of Interests	Outcomes of interest agreed prior to undertaking research Protocols and analysis plans made available to peers for scrutiny Conflicting interests which could engender bias made known to public and peers
Other questionable research practices	Practices including deciding to collect more data after inspecting results, selective rounding of p-values, selective reporting of dependent variables.	Registered Reports Research Registry	Methods are agreed prior to publication. Incentive to generate results which favour publication removed Protocol and analysis plan made available to peers for scrutiny
Undisclosed conflicts of interest	Researchers may have, or could be perceived to have, vested interest in obtaining certain outcome in their results.	Mandatory Declaration of Interests	Researchers compelled to made comprehensive statement of their pecuniary interests, gifts and hospitality received and non-pecuniary interests
Non-replicable research	Results unable to be replicated, either because of insufficient information to reproduce methods or because of biases in original study (including problems in this table) mean work not reproduced when attempted.	Research Registry	Adequate study documentation made available such that study can be repeated or analyses repeated.

**Box 1.** Development of the authors’ strategy to achieve further improvements in medical research.

The concept of a campaign, with specific objectives to improve health research arose from a presentation made to the EBMLive conference in Oxford in July 2019. The conference organisers facilitated a further session to discuss the concept further, chaired by Georgia Richards. An online survey was distributed among attendees of the conference session. Survey results were used to inform selection of three demands. Drafts of a statement were prepared by Stephen Bradley, Peter Gill and Georgia Richards and distributed among conference attendees, who then provided feedback and became signatories. A draft statement was made available (https://osf.io/k3w7m/) in October 2019 and publicised via twitter (@TA_Declaration). The feedback received informed the second major revision of the declaration, which, following consultation with signatories, was made available in January 2020. Signing the statement and/or providing feedback is possible by completing this form: https://docs.google.com/forms/d/1wHWcw77fFtvvvY2tJpNrzflt3HE0tF8xvFOL3U55RV4/

#### Mandatory declaration of interests

To establish trust in the objectivity of research, researchers must become more open about conflicts or declarations of interests. The brief voluntary statements in publications are not sufficient to meet this expectation. A fully accessible database of interests should be established, with firm expectations of accurate, up-to-date and comprehensive disclosure from all researchers, doctors, institutions and patient advocacy groups. Such disclosures should feature both monetary payments, benefits such as travel, hospitality and conference fees, and non-monetary interests, such as memberships of committees. Researchers’ declaration of interests should be required by all academic institutions, funders and journals. The Open Research and Contributor ID (ORCID) system already catalogues the identities of over 7 million researchers. Hence, ORCID could be an option to index researchers’ interests.^79^

#### Registered reports

Registered reports are a publication format that permits authors to submit their proposed methods and analysis plan to a journal prior to conducting the research. If the journal accepts the proposal, it commits to publication as long as the research is satisfactorily conducted, regardless of the findings.^80^ This model can help limit the impacts of publication bias and other reporting biases. Registered reports also curtail both the ability of authors to undertake (or peer reviewers to request) HARKing or p-hacking, and reduce the incentive to do so. Underpowered research or questionable methodological or statistical decisions can be identified and addressed through peer-review prior to study conduct. Since journals commit to publication upon review of study plans, rather than finished papers, registered reports may reduce the incentive for authors to ‘spin’ and for reviewers to request such embellishments.

Authors are not restricted from undertaking additional exploratory analyses, but registered reports help clarify that such analyses are exploratory and not based on prior hypotheses. Null findings are more likely to be published through registered reports than traditional formats and registered reports are cited just as often as conventional papers.^81,82^

At the time of writing, registered reports were accepted by 225 journals, which included only 68 (1.3%) of the 5250 journals indexed in MEDLINE (Supplement 3).^80^ A precursor to the registered report format was introduced by the *Lancet* over 20 years ago, but was discontinued with the warning that some studies deviated substantially from pre-specified outcomes and analyses.^83^ This experience suggests that consistent evaluation of registered reports and support for authors to use the format is required. Their successful adoption in other scientific disciplines suggests medical journals could adopt this format in much greater numbers than they currently do. Pressure from funders, authors, readers and editorial board members could help support journals to make this transition.

#### Comprehensive research registration and publication

To facilitate reproducibility of research findings and to assess the plausibility of scientific claims, it is essential that documentation, including protocols and analysis plans, are made available to peers. Making all study findings available is the only way to address publication bias. It is also a matter of fairness that research which is paid for by public or charitable funding, and upon which important healthcare decisions may be made, is made available for anyone to view.

For all publicly funded research, not just trials, comprehensive documentation including protocols, statistical analysis plans, statistical analysis code, raw or appropriately de-identified summary data, and results should be available on a World Health Organization-affiliated open access registry. In theory, the Food and Drug Administration already requires that protocols and statistical analysis plans for clinical trials are publicly shared.^59^ Obtaining widespread compliance with this principle for most types of study would represent a significant, but achievable, advance in transparency and fairness. Funders should require that study documentation is made openly available, while governmental and national research institutions could support the development, or nomination of, an appropriate open platform where anyone can find comprehensive information about publicly funded research.

Important principles such as the range of documentation that should be shared and to what level of detail would need to be established. However, we believe that establishing the expectation that sufficient information should be available for research to be adequately appraised could be an important milestone in achieving greater transparency and reproducibility.

## Implementation

Implementing the proposed measures would likely impose financial and opportunity costs. Registered reports could create additional workload for journals and peer reviewers, and shift substantial work to earlier in the lifecycle of a project which may have funding and resource implications for research teams. Additionally, establishing a register of interests and a dedicated research repository would require investment from research funders and regulators, as well as the participation of researchers and industry. We believe the measures are proportionate, particularly in the context of the profits generated by the publishing industry from research^84^ and the large sum of public resources invested to produce those findings.^1^ The implementation of registers elsewhere, even in highly decentralised healthcare settings like the USA, is a proof of concept for the viability on other settings. While researchers might be reluctant to accept additional administrative burdens, depositing interests and study data in central and accessible locations could help reduce duplicative reporting requirements.

Important stakeholders such as research funders, regulators and journal editors are likely to have the most influence in achieving change. Yet the experience of inconsistent compliance with existing requirements^85–87^ suggests that cultural change is also vital. Campaigns that raise awareness and expectations of transparency from the public^88–90^ and legislators^91^ along with social pressure from peers^92^ are likely to help with embedding improvements in research culture.

## Conclusions

Significant progress is required to satisfy reasonable expectations that medical research is trustworthy, reproducible and represents value for money. The proposed strategy comprising mandatory registration of potentially competing interests, registered reports and requiring all publicly funded research is registered can be readily conveyed to policy makers and rapidly implemented. These ideas are not novel and we do not claim that they would solve all problems in medical research. But, while such profound problems persist in medical research, we believe that it is time to implement simple measures to achieve greater transparency, reduce reporting biases and deter poor methodological practices.

## Supplemental Material

sj-pdf-1-jrs-10.1177_0141076820956799 - Supplemental material for Reducing bias and improving transparency in medical research: a critical overview of the problems, progress and suggested next steps Click here for additional data file. Supplemental material, sj-pdf-1-jrs-10.1177_0141076820956799 for Reducing bias and improving transparency in medical research: a critical overview of the problems, progress and suggested next steps by Stephen H Bradley, Nicholas J DeVito, Kelly E Lloyd, Georgia C Richards, Tanja Rombey, Cole Wayant and Peter J Gill in Journal of the Royal Society of Medicine

## Declarations

**Ethical approval:** Not applicable.

**Guarantor:** SHB.

**Contributorship:** PJG originated the idea for the paper. KEL designed the search strategy and undertook the searches. TR determined the number of journals listed in MEDLINE which accept registered reports (Supplement 2). SHB prepared the initial draft with all authors contributing to subsequent revisions. All authors provided comments and reviewed the manuscript.

**Acknowledgements:** The majority of the authors met at the EBMLive conference and wish to thank the McCall MacBain Foundation funding TR and SHB to attend as Doug Altman scholars. The authors would also like to thank all of those who have contributed to, and provided feedback for the Declaration to Improve Biomedical and Health Research.

**Provenance:** Not commissioned; peer-reviewed by Mandy Payne.
