# Supplementary material for: Reducing bias and improving transparency in medical research: a critical overview of the problems, progress and suggested next steps
Source: J R Soc Med. 2020 Nov 10;113(11):433–43. doi: 10.1177/0141076820956799 (PMC7673265; doi:10.1177/0141076820956799)
Supplement: sj-pdf-1-jrs-10.1177_0141076820956799 - Supplemental material for Reducing bias and improving transparency in medical research: a critical overview of the problems, progress and suggested next steps [file sj-pdf-1-jrs-10.1177_0141076820956799.pdf]

Supplement 1

Ovid MEDLINE(R) - 1996 to January Week 2 2020

1. open science.mp. [mp=title, abstract, original title, name of substance word, subject heading word, floating sub-heading word, keyword heading word, organism supplementary concept word, protocol supplementary concept word, rare disease supplementary concept word, unique identifier, synonyms]

2. open research.mp. [mp=title, abstract, original title, name of substance word, subject heading word, floating sub-heading word, keyword heading word, organism supplementary concept word, protocol supplementary concept word, rare disease supplementary concept word, unique identifier, synonyms]

3. research quality.tw.

4. medic*.mp. [mp=title, abstract, original title, name of substance word, subject heading word, floating sub-heading word, keyword heading word, organism supplementary concept word, protocol supplementary concept word, rare disease supplementary concept word, unique identifier, synonyms]

5. health*.mp. [mp=title, abstract, original title, name of substance word, subject heading word, floating sub-heading word, keyword heading word, organism supplementary concept word, protocol supplementary concept word, rare disease supplementary concept word, unique identifier, synonyms]

6. 1 or 2 or 3

7. 4 or 5

8. 6 and 7

Search results: 624 papers

Ovid EMBASE - 1996 to 2020 Week 02

1. open science.mp. [mp=title, abstract, heading word, drug trade name, original title, device manufacturer, drug manufacturer, device trade name, keyword, floating subheading word, candidate term word]

2. open research.mp. [mp=title, abstract, heading word, drug trade name, original title, device manufacturer, drug manufacturer, device trade name, keyword, floating subheading word, candidate term word]

3. research quality.tw.

4. medic*.mp. [mp=title, abstract, heading word, drug trade name, original title, device manufacturer, drug manufacturer, device trade name, keyword, floating subheading word, candidate term word]

5. health*.mp. [mp=title, abstract, heading word, drug trade name, original title, device manufacturer, drug manufacturer, device trade name, keyword, floating subheading word, candidate term word]

6. 1 or 2 or 3

7. 4 or 5

8. 6 and 7

Search results: 1168 papers

Ovid PsycINFO - 2002 to January Week 1 2020

1. open science.mp. [mp=title, abstract, heading word, table of contents, key concepts, original title, tests & measures, mesh]

2. open research.mp. [mp=title, abstract, heading word, table of contents, key concepts, original title, tests & measures, mesh]

3. research quality.tw.

4. medic*.mp. [mp=title, abstract, heading word, table of contents, key concepts, original title, tests & measures, mesh]

5. health*.mp. [mp=title, abstract, heading word, table of contents, key concepts, original title, tests & measures, mesh]

6. 1 or 2 or 3

7. 4 or 5

8. 6 and 7

Search results: 202 papers

CINAHL (EBSCOhost)

TX "open science" OR TX "open research" OR TI "research quality" OR AB "research quality" AND TX (medic* OR health*)

Limiters - English Language; Exclude MEDLINE records

Search modes - Boolean/Phrase

Search results: 293 papers

Initiative/Organisation	Description	URL
AllTrials	Campaign to ensure all clinical trials are registered and published. Highlights problem of publication bias, e.g. through 'unreported clinical trial of the week'	https://www.alltrials.net/
Centre for Open Science (COS)	Organisation dedicated to 'increase the openness, integrity and reproducibility of scientific research'	https://cos.io/
Centre for Evidence Based Medicine	University of Oxford based centre which develops, promotes and disseminates better evidence for healthcare, including contributing to the EBMLive conference	https://www.cebm.net/
ClinicalStudyDataRequest.com (CSDR)	Consortium of clinical study Sponsors/Funders which facilitates access to patient-level data from clinical studies.	https://clinicalstudydatarequest.com
cOALition S (Plan S)	Formed by national research funding organisations, with the support of the European Commission and the European Research Council (ERC) to achieve greater open access publication of research	https://www.coalition-s.org/
Collaborative Approach to Meta Analysis and Review of Animal Data from Experimental Studies	Provides a supporting framework for groups involved in the systematic review and meta-analysis of data from experimental animal studies.	http://www.dcn.ed.ac.uk/camarades/
Cochrane	Charitable organisation formed to organise medical research findings to facilitate evidence-based choices about health interventions involving health professionals, patients and policy makers	https://www.cochrane.org/
Enhancing the QUAlity and Transparency Of health Research (EQUATOR) Network	International initiative which aims to improve the reliability of published health research by promoting transparent and accurate reporting and wider use of robust reporting guidelines.	https://www.equator-network.org/
European Open Science Cloud (EOSC)	On-line platform which aims to facilitate curation of open science data	https://ec.europa.eu/research/openscience/index.cfm?pg=open-science-cloud
European Quality in Preclinical Data (EQIPD)	Consortium which aims to improve transition from preclinical to clinical testing and drug approval by establishing common guidelines to strengthen the robustness, rigor and validity of research data.	https://quality-preclinical-data.eu

Evidence Based Research Network (EBRNetwork)	European network established to support evidence based clinical research, particularly the need to use systematic reviews when planning new studies and when placing new results in context.	http://ebrnetwork.org/
Ensuring value in health-related research (EVIR)	Collaboration of funders which aims to increase value of health research	https://www.thelancet.com/journals/lancet/article/PIIS0140-6736(18)30464-1/fulltext
Evidence-Based RESearch (evbres)	European Union funded project which promotes an evidence based research approach in clinical research	https://evbres.eu
FAIR Data Principles	Guiding principles to make data Findable, Accessible, Interoperable, and Reusable	https://www.force11.org/group/fairgroup/fairprinciples
German Network for Evidence Based Medicine (EBM Netzwerk)	Promotes the quality of patient care and disease prevention by applying the principles of evidence-based healthcare	https://www.ebm-netzwerk.de/de
HealthWatch UK	UK charity that promotes science and integrity in healthcare and health research	https://www.healthwatch-uk.org/
The Hong Kong Principles for assessing researchers	Principles agreed at the 6th World Conference on Research Integrity which aim to bring considerations of trustworthiness, rigour and transparency to the assessment of research	https://www.wcrif.org/guidance/hong-kong-principles
medRxiv	Server for preliminary versions of clinical research articles (preprints) so that they can be made available prior to publication	https://www.medrxiv.org/
Open Science Framework (OSF)	On-line platform which facilitates open sharing and preregistration of research.	https://osf.io/
Open Science Badges	Badges appended to publications to acknowledge and incentivise open science practices	https://cos.io/our-services/open-science-badges/
QUEST (Quality, Ethics, Open Science,Translation) Center for Transforming Biomedical Research	Based in Berlin Institute of Health, hosts three research groups and activities include meta research to identify measures for improving research practice	https://www.bihealth.org/en/research/quest-center/mission-approaches/
Preclinical Trials	International register of preclinical trial protocols	https://www.preclinicaltrials.eu/
Registered Reports	Publication format aimed at reducing reporting bias, supported by the Centre for Open Science and other institutional/individual Open Science campaigners	https://cos.io/rr/

The Reproducibility Project: Cancer Biology	Collaboration between Science Exchange & Center for Open Science, replicating experimental results from high-profile papers in cancer biology published between 2010-2012	https://osf.io/e81xl/wiki/home/
REWARD Alliance	Formed following the 2014 Lancet Series on Waste in Research, and exists to share and exchange documentation, information, and resources to help increase the value of research and reduce waste in research.	http://rewardalliance.net/
San Francisco Declaration on Research Assessment (DORA)	Initiative which calls for improvement in how research quality is evaluated	https://sfdora.org/
Sense About Science	Campaigning charity that challenges misrepresentation of science and evidence and advocates for increased openness and honesty about research findings	https://senseaboutscience.org/
Sunshine UK	Voluntary register of doctors' declared interests	http://www.whopaysthisdoctor.org/
Transparency and Openness Promotion (TOP) guidelines	Graded standards for openness and transparency of research	https://cos.io/top/
TranspariMED	Campaigning group which advocates for registration and full reporting of clinical trials	https://www.transparimed.org/
Trial Forge	Project which aims to collate and share ways to improve all the processes involved in clinical trials	https:trialforge.org
The Trials Tracker Project	Trackers created by EBM Datalab (University of Oxford) which monitor trial reporting performance of pharmaceutical companies, universities, funders, sponsors, and other organisations	http://trialstracker.net/
Vivli	Research data sharing platform	https://vivli.org/
Yale University Open Data Access (YODA) Project	Advocates for the responsible sharing of clinical research data and has developed a model to make data available to researchers	https://yoda.yale.edu/
